# Local versus general anesthesia for transcatheter aortic valve implantation (TAVR) – systematic review and meta-analysis

**DOI:** 10.1186/1741-7015-12-41

**Published:** 2014-03-10

**Authors:** Georg M Fröhlich, Alexandra J Lansky, John Webb, Marco Roffi, Stefan Toggweiler, Markus Reinthaler, Duolao Wang, Nevil Hutchinson, Olaf Wendler, David Hildick-Smith, Pascal Meier

**Affiliations:** 1 The Heart Hospital, University College London Hospitals, London, UK; 2 Division of Cardiology, Yale Medical School, New Haven, CT, USA; 3 Department of Cardiology, St. Paul’s Hospital, University of British Columbia, Vancouver, BC, Canada; 4 Department of Cardiology, University Hospital Geneva HUGE, Geneva, Switzerland; 5 Department of Cardiology, Kantonsspital Luzern, Lucerne, Switzerland; 6 Department of Medical Statistics, London School of Hygiene and Tropical Medicine London, London, UK; 7 Division of Anesthesiology, Brighton and Sussex University Hospital, Brighton, UK; 8 Department of Cardiothoracic Surgery, King’s College Hospital, London, UK; 9 Division of Cardiology, Brighton and Sussex University Hospital, Brighton, UK

**Keywords:** TAVR, Local anesthesia, General anesthesia, Aortic stenosis

## Abstract

**Background:**

The hypothesis of this study was that local anesthesia with monitored anesthesia care (MAC) is not harmful in comparison to general anesthesia (GA) for patients undergoing Transcatheter Aortic Valve Implantation (TAVR).

TAVR is a rapidly spreading treatment option for severe aortic valve stenosis. Traditionally, in most centers, this procedure is done under GA, but more recently procedures with MAC have been reported.

**Methods:**

This is a systematic review and meta-analysis comparing MAC versus GA in patients undergoing transfemoral TAVR. Trials were identified through a literature search covering publications from 1 January 2005 through 31 January 2013. The main outcomes of interest of this literature meta-analysis were 30-day overall mortality, cardiac-/procedure-related mortality, stroke, myocardial infarction, sepsis, acute kidney injury, procedure time and duration of hospital stay. A random effects model was used to calculate the pooled relative risks (RR) with 95% confidence intervals.

**Results:**

Seven observational studies and a total of 1,542 patients were included in this analysis. None of the studies were randomized. Compared to GA, MAC was associated with a shorter hospital stay (-3.0 days (-5.0 to -1.0); *P* = 0.004) and a shorter procedure time (MD -36.3 minutes (-58.0 to -15.0 minutes); *P* <0.001). Overall 30-day mortality was not significantly different between MAC and GA (RR 0.77 (0.38 to 1.56); *P* = 0.460), also cardiac- and procedure-related mortality was similar between both groups (RR 0.90 (0.34 to 2.39); *P* = 0.830).

**Conclusion:**

These data did not show a significant difference in short-term outcomes for MAC or GA in TAVR. MAC may be associated with reduced procedural time and shorter hospital stay. Now randomized trials are needed for further evaluation of MAC in the setting of TAVR.

## Background

Transcatheter Aortic Valve Implantation (TAVR) is a rapidly evolving procedure for patients with severe aortic stenosis. TAVR was initially designed as a less invasive technique for patients who were unsuitable or at high risk for conventional valve surgery [1,2]. With emerging new valve technologies and improving operator experience, TAVR is likely to become an alternative option for patients at intermediate risk in the near future. In 2009, 4,498 patients underwent TAVR in Europe and the numbers were rapidly growing to 18,372 in 2011 [1,3]. In Germany, for example, TAVR is now used for approximately 50% of patients ≥75 years of age [4]. Currently, the vast majority of TAVR procedures are performed under general anesthesia (GA). GA is usually provided by an anesthetist experienced in managing patients undergoing conventional cardiac surgery. There are considerable regional differences, with nearly 100% of cases done under GA in the US, >80% in the UK and 66% in France [3,5]. Initial small observational studies suggested that monitored anesthesia care (MAC) may be feasible and safe [6,7]. MAC was defined as cardiovascular and respiratory monitoring of the patient by a qualified anesthesiologist who may or may not be administering concomitant sedation. For endovascular aortic aneurysm repair, MAC has proven to be beneficial in a large population of high risk patients [8]. Therefore, the impact of MAC versus GA on outcomes for other interventional endovascular procedures, such as TAVR, which are currently performed predominantly under GA, should also be assessed. It is well known that GA and, in particular, mechanical ventilation may be complicated by pneumonia, hemodynamic compromise and the need for extensive catecholamine use [9,10]. Further, prolonged intensive care or in-hospital stays are associated with increased risk of nosocomial infections and mortality [11]. This study intended to test the hypothesis that MAC is equally safe as GA for TAVR.

## Methods

The study was performed according to the preferred reporting items for systematic reviews and meta-analyses (PRISMA) guidelines for meta-analyses (Additional file 1) [12,13]. Planning and study design were done by two authors (GF, PM) including creation of an electronic database with variables of interest. The main outcome variables of interest and search strategy (databases, sources for unpublished data) were defined in a strategy outline.

### Search strategy

We searched EMBASE, PubMed, MEDLINE, BIOS and ISI Web of Science for manuscripts published from 1 January 2005 through 31 January 2013. In addition, abstract lists and conference proceedings from the 2006 to 2012 scientific meetings of the American College of Cardiology, the European Society of Cardiology, the Symposium on Transcatheter Cardiovascular Therapeutics, the American Heart Association, and the World Congress of Cardiology were searched. We also considered published review articles, editorials and internet-based sources of information [14-18] to assess potential information on studies of interest. Reference lists of selected articles were reviewed for other potentially relevant citations. No language restriction was applied. The detailed search syntax for the database Medline is shown in Additional file 2. The syntax for other databases was similar but was adapted where necessary. In the absence of any prospective randomized studies, only non-randomized observational studies could be included.

### Study selection

**Figure 1:**
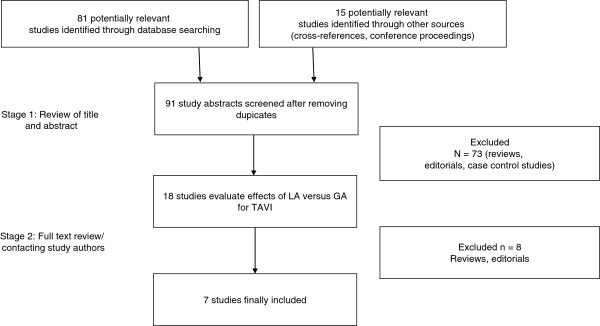
Flow chart of study selection process.

In a two-step selection process, the titles and abstracts of all citations were reviewed independently by two researchers (PM, GF) to identify potentially relevant studies. In a second step, the corresponding publications were reviewed in full text to assess if studies met the following inclusion criteria: MAC for TAVR procedures and a GA control group (Figure 1).

#### Data extraction

Relevant information from the articles, including baseline clinical characteristics of the study population and outcome measures, were extracted using the prepared standardized extraction database; we focused on unadjusted and observed outcomes.

### Outcome measures

Baseline variables and clinical and angiographic data were extracted and compared. Main outcome variables of interest were overall 30-day mortality, cardiac-/procedure-related mortality, in-hospital or procedure-related complications, stroke, myocardial infarction, vascular complications, procedural success, acute kidney injury, procedural time and in-hospital stay. The endpoint definitions are described in Additional file 3.

### Data synthesis and analysis

Data analysis was performed on an intention-to-treat analysis, that is, patients who converted from one approach to the other (MAC to GA) during the procedure were considered to be in the group they were originally assigned to. Data of the selected non-randomized observational studies were combined to estimate the pooled effect (risk ratio, RR) of local versus GA. Calculations were based on a DerSirmonian and Laird random-effects model, using an asymptomatic approach [19]. This model assumes that the true effects vary between studies for unknown reasons. The primary summary measure usually reported is the estimated average effect across studies [20]. Continuity correction was used when no event occurred in one group to allow calculation of a RR [21]. Heterogeneity among trials was quantified with Higgins’ and Thompson’s I^2^[22]. *I*^*2*^ can be interpreted as the percentage of variability due to heterogeneity between studies rather than sampling error. An *I*^*2*^ > 50% was considered as at least moderate heterogeneity. Weighted average incidence of events was calculated based on a random-effect analysis using a Freeman-Tukey double arcsine transformation and the inverse variance method [23]. We present our primary result estimates of the average effect across studies with 95% confidence intervals in brackets. We did not test for publication bias or small study effects due to the small number of studies included in this analysis. We have performed a sensitivity analysis excluding “gray literature” data (not yet published in peer-reviewed literature). We also performed the analyses with an exact permutation test for meta-analyses. All analyses were performed with “R”, version 2.15.1 (packages “meta”, “metafor”) R Foundation for Statistical Computing, Vienna, Austria.

## Results

### Description of included studies and baseline characteristics

A total of 79 articles were reviewed and 7 studies, including 1,542 patients, satisfied the predetermined inclusion criteria (Figure 1) [5-7,24-27]. Studies using only general anesthesia or only MAC were not considered. All studies used either a transfemoral or transaxillary approach for TAVR. Additional file 4 shows the valve types used: three studies used predominantly or exclusively the CoreValve (Medtronic, Minneapolis, MN, USA) [5,7,24], three studies used predominantly the Edwards SAPIEN or SAPIEN XT valves (Edwards Lifesciences, Irvine, CA, USA) [6,25,26], one study did not describe which valve type was used [27]. Table 1 shows the baseline characteristics in the different studies. In two studies, the overall risk scores (logistic EuroSCORE and/or Society of Thoracic Surgeons (STS) score where higher for the GA group [7,27], in one study, the risk score was significantly higher for MAC [25], while the remaining studies did not find a statistically significant difference (Table 1). Additional file 5 describes the decision-making process regarding GA versus MAC. None of the included studies was randomized.

**Table 1:** Differences in baseline characteristics between MAC and GA

**Study**	**Yamamoto**		**Motloch**		**Dhedin**		**Ben-Dor**		**Behan**		**Linke**		**Covello**	
	**MA**	**GA**	**MAC**	**GA**	**MAC**	**GA**	**MAC**	**GA**	**MAC**	**GA**	**MAC**	**GA**	**MAC**	**GA**
n	130	44	41	33	34	91	42	27	9	3	547	449	42	27
Logistic EuroScore	22.0*	26.6*	NA	NA	23.6	24	40.1*	28.1*	21.8	22.9	**	**	27.3	22.9
STS score (mortality)	11.2	14.3	20.8	16.5	9.2*	14*	NA	NA	NA	NA	NA	NA	NA	NA
Prior CVA/TIA	10.1%	11.1%	14.6%	24.2%	12%	11%	30%*	9.1%*	11%	33%	NA	NA	19%	15%
CAD	10.1%	11.1%	43.9%	42.4%	44%	52%	55.7%	45.5%	NA	NA	**	**	57%	44%
Renal dysfunction (CrCl >60 ml/minute)	68.5%	63.6%	NA	NA	56%	44%	37.1%	50%	11%	0%	NA	NA	NA	NA
Age	83.7	84.7	82.6	83.4	83.5	83	84.1	83.7	80	83	**	**	79.5	77.6
Females	60.5%	53.3%	65.9%	45.5%	47%	50%	58.5%	63.6%	33%	33%	**	**	NA	NA
Diabetes	25.6%	13.3%	29.3%	27.3%	23%	19%	30%	31.8%	NA	NA	**	**	33%	30%
Hypertension	81.4%	73.3%	82.5%	75.8%	74%	64%	90%	95.5%	NA	NA	NA	NA	NA	NA
COPD	23.3%	24.4%	9.8%	12.1%	15%	30%	14.3%	9.1%	33%	33%	NA	NA	69%	37%
EF	50.4%*	45.1%*	53.6%	54.8%	57%	50%	52.7%	55.2%	NA	NA	NA	NA	NA	NA
AVA (cm^2^)	0.67	0.72	0.6	0.6	0.42	0.38	0.63	0.65	NA	NA	NA	NA	NA	NA

A, not available; AVA aortic valve area, CAD coronary artery disease, COPD, chronic obstructive pulmonary disease; CVA/TIA, cerebrovascular accident/transient ischemic attack; EF ejection fraction, GA, general anesthesia; MAC, monitored anesthesia care; STS, Society of Thoracic Surgeons score; *significant. **No statistically significant difference according to authors, numbers not available.

#### Overall mortality

**Figure 2:**
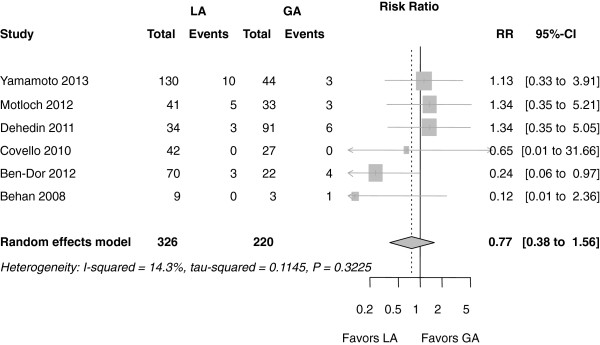
**Forest plot of risk ratios for 30-day mortality.** Markers represent point estimates of risk ratios, marker size represents study weight in random-effects meta-analysis. Horizontal bars indicate 95% confidence intervals. CI, confidence interval; RR, risk ratio.

The average 30-day mortality rate was 4.2% (1.5 to 9.2%) in the MAC group and 5.4% (1.1 to 11.7%) in the GA group. This difference was not statistically different between the groups (RR 0.77 (0.38 to 1.56); *P* = 0.460) (Figure 2).

The cardiac and procedure-related mortality was not significantly different either (RR 0.90 (0.34 to 2.39); *P* = 0.830) (Additional file 6).

#### Procedural outcomes

**Figure 3:**
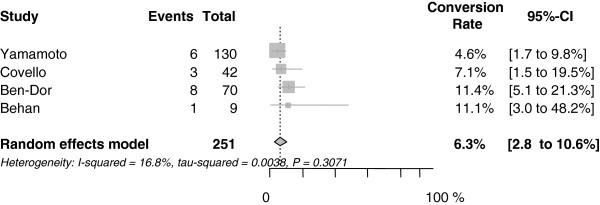
**Forest plot for conversion rate from MAC to GA.** CI, confidence interval.

The conversion rate from MAC to GA was 6.3% (2.8 to 10.6%) or 18 out of 251 patients (Figure 3). Additional file 7 describes the reasons for the switch.

Vascular complications were observed in 8.4% (4.8 to 12.8%) or 18 out of 205 patients in the MAC group and in 15.6% (5.8 to 28.5%) or 31 out of 168 patients in the GA group. However, the difference was not statistically significant (RR 0.66 (0.35 to 1.25): *P* = 0.210) (Additional file 8).

Also, the procedural success (as defined in the individual trials) was very similar in the study groups (94.6% (90.8 to 97.5%) in the MAC group (193 of 205 patients) and 96.4% (91.3 to 99.6%)) in the GA group (160 of 168 patients), (RR 0.98 (0.91 to 1.06); *P* = 0.620) (Additional file 9).

**Figure 4:**
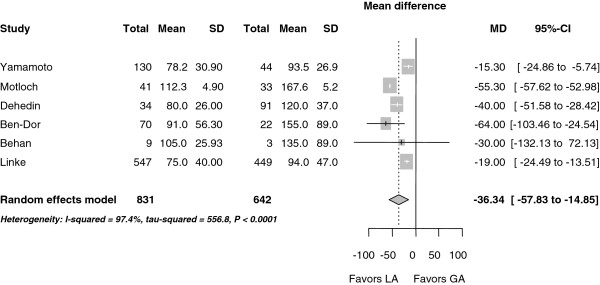
**Forest plot for procedural time (minutes).** CI, confidence interval.

MAC was associated with a significantly shorter procedure time compared to GA, (MD -36 minutes, (-58.0 to -15.0 minutes); *P* <0.001) (Figure 4). There was considerable heterogeneity among the trials with an I^2^ of 97.4%.

### Post-procedural outcome

**Figure 5:**
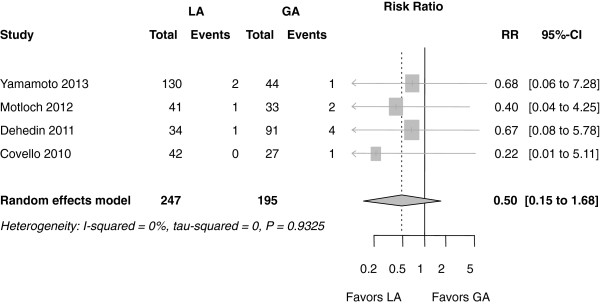
**Forest plot of risk ratios (RR) for stroke.** CI, confidence interval; RR, risk ratio.

On average, the stroke rate was 1.2% (0.1 to 3.3%) in the MAC group (4 of 247 patients) and 3.8% (1.3 to 7.3%) in the GA group (8 of 195 patients). Even though this stroke rate was numerically lower in the MAC group, it was not statistically significant (RR 0.50 (0.15 to 1.68); *P* = 0.460) (Figure 5).

The rate of myocardial infarction was not different between the study groups (2 of 247 patients in the MAC and 1 in 195 patients in the GA group; RR 1.06 (0.20 to 5.54): *P* = 0.950) (Additional file 10).

Post-interventional acute kidney injury did not differ between the groups (28 of 247 patients in the MAC and 19 of 195 patients in the GA group; RR was 0.88 (0.50 to 1.55); *P* = 0.650) (Additional file 11).

**Figure 6:**
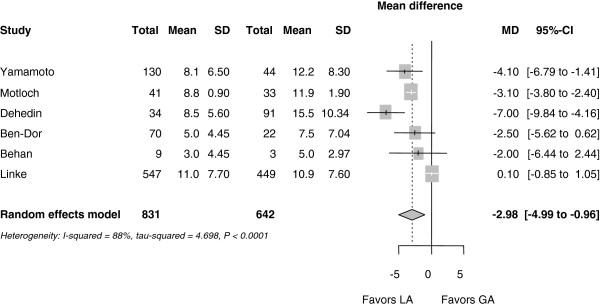
**Forest plot for hospital stay (days).** CI, confidence interval.

MAC was associated with significantly shorter hospital stay (MD -3.0 days (-4.99 to -0.96 days); *P* = 0.004) (Figure 6). There was considerable heterogeneity among the trials with an I^2^ of 88%.

The occurrence of peri-operative sepsis did not differ significantly between the study groups (13 of 117 patients in the MAC and 15 of 151 patients in the GA group; RR 1.23 (0.59 to 2.53); *P* = 0.580) (Additional file 12).

Only one study reported on chest infections. Covello *et al*. found a pneumonia rate of 8% after GA and 0% after MAC.

#### Sensitivity analyses

We have repeated the key analyses excluding the study of Linke *et al*. which has only been published as an abstract [5]. Since this abstract contained limited information it was only used for the calculation of the following endpoints:

Procedure duration was reduced by 41 minutes (95% CI 63 to 18 minutes; *P* <0.001] on average in the MAC group (the reduction was 36 minutes if Linke *et al*. was included). The duration of hospital stay was reduced by 3.8 days (5.3 to 2.3 days; *P* <0.001) with MAC (the reduction was 3.0 days, if Linke *et al*. was included).

Using the exact permutation test approach, the results were nearly similar: the RR for mortality was 0.77 (0.37 to 1.60); *P* = 0.656. The RR for cardiac and procedure-related mortality was RR 0.90 (0.34 to 2.40); *P* = 0.375. For stroke, it was 0.50 (0.15 to 1.68); *P* = 0.125. For vascular complications, the RR was 0.66 (0.35 to 1.25): *P* = 0.500.

For sepsis, it was 1.23 (0.59 to 2.53); *P* = 0.500. For myocardial infarction, it was 1.05 (0.20 to 5.54): *P* = 0.999. For acute kidney injury, the RR was 0.88 (0.50 to 1.55); *P* = 0.875.

Further, we have excluded the very small study by Behan *et al*. The results were very similar: for mortality the RR was 0.86 (0.43 to 1.70); *P* = 0.662, for procedural cardiac death, it was 0.96 (0.35 to 2.64); *P* = 0.934.

## Discussion

This is the first meta-analysis that compared the outcome of MAC versus GA in patients undergoing transfemoral TAVR. It is based on non-randomized data exclusively. Mortality and safety endpoints did not significantly differ between the two approaches. Procedural time and in-hospital stay were significantly reduced with MAC. The need for conversion from MAC to GA was infrequent.

Interestingly, the very first TAVR procedure, done over a decade ago, was performed under MAC. With the decrease in sheath sizes and better closure devices, an increasing number of operators might wish to switch to a predominantly percutaneous approach under local anesthesia. Robust data on safety and risk of this approach are therefore needed.

### GA versus MAC

GA is generally the preferred option for patients undergoing any major surgical interventions [28]. However, GA itself carries a procedural mortality risk that averages 0.03 deaths per 1,000 patients, with even more of a pronounced risk in open heart surgery and in a higher risk population, such as the population currently considered for TAVR [29]. While this risk is clearly justified for conventional cardiac surgery, its role can be challenged for TAVR [27,30]. GA in patients with severe aortic stenosis may even be associated with a particularly increased peri-procedural risk [31]. However, GA has certainly multiple advantages for the operator:

• It enables real-time transesophageal echocardiography (TEE) which might especially be helpful for appropriate valve sizing and positioning, and for prompt recognition of complications such as aortic dissection, tamponade and valve embolization [32]. However, valve positioning is mainly guided by fluoroscopy [33]. Echocardiography does not seem to relevantly reduce contrast dye use. Two studies where no TEE was used in the GA arm reported on contrast dye use, and did not find a relevant difference [7,25]. Alternatively, intracardiac echocardiography, or even transthoracic echocardiography could be used for MAC. Furthermore, although rare, TEE itself can lead to serious complications, such as esophageal hematoma or rupture [34].

• GA may provide more stable conditions. Indeed, it prevents the patient from moving, especially during the critical phase of valve deployment under rapid pacing. During this period with a reduced cardiac output, patients with MAC might become disorientated which may provoke movements. On the other hand, our data show that patients with GA were more likely to need catecholamine support, as compared to MAC [6]. Indeed, MAC can achieve similarly stable conditions.

• GA allows a quick conversion to bail-out surgery in case of peri-procedural complications. However, a conversion to surgery is an infrequent event [6,7,26]. Furthermore, although only a few observational studies report these data, conversion from MAC to GA appears to be safe if operators are prepared for this event. So far, there are no data indicating an increased mortality risk after conversion from MAC to GA [7,24-26].

MAC, on the other hand, has the advantage of shorter procedure durations and a prompter recovery period with shorter hospital stay [35]. A shorter hospital stay decreases the risk for nosocomial infections and other complications associated with a hospital stay [35]. Indeed, nosocomial infection represents a significant problem; it is the eighth leading cause of death in the US [36]. Moreover, mechanical ventilation is directly related to an increased risk for pneumonia, especially in an elderly population [37]. Infections should be avoided as far as possible, also, because of the risk of aortic valve prosthesis endocarditis [38].

Mortality and stroke rates were numerically lower for MAC compared to GA, but this difference was not statistically significant. Whether this was due to a lack of statistical power or whether these differences are indeed simply a play of chance is unknown. We also have to consider that the heterogeneous definitions for most endpoints are a major limitation. Theoretically, MAC may allow earlier recognition of complications compared to non-responsive patients under GA (for example, stroke, retroperitoneal bleed). GA may also result in pronounced hypotension. Whether this would actually translate into an earlier and more effective treatment and an improved outcome remains speculative.

Notably, procedural time (predominantly defined as the span of time between the patient entering until the patient leaving the cath lab) and the total hospital stay were significantly shorter for MAC. The difference in procedural time was probably predominantly driven by the additional need for anesthesia induction and weaning/extubation after the procedure for patients undergoing GA. For the patient, only the actual procedure time really matters, but the total cath-lab time can have an impact on resource use and costs. However, only one study discriminated between total procedural time and interventional time, both parameters were in favor of MAC [6]. Interestingly, this study was the only one assessing health economic aspects. The authors found a 63.4% reduction in cath-lab related costs with MAC [6]. This was mainly due to the reduced number of staff needed and the shorter use of the cath-lab [6]. However, we have to be aware that this was not a formal cost-effectiveness analysis; it did not consider utility or the costs beyond the staff costs related to the procedure itself. Procedure-related costs account for only approximately 50% of the total costs for a TAVR procedure, which is approximately €40,000 (approximately $53,400) [39]. Some studies found a reduced need of “high dependency care” after MAC [6,24-26]. Importantly, intensive care stay is also a major contributor to health care expenditure, especially if prolonged mechanical ventilation is necessary [40]. Considering that our study did not find relevant differences in outcomes and MAC is likely to reduce the resource need, MAC may be the overall cheaper option. However, this very much depends on local factors and remains speculative at this stage. A prospectively planned cost-effectiveness analysis, optimally linked to a randomized trial comparing MAC versus GA would be needed to shed light on the impact of MAC and GA on costs.

It is not clear why GA patients had longer hospital stays. This may be due to a prolonged post-procedure recovery period or simply due to differences in local protocols.

MAC appears to be safe and cost-effective and might even yield an improved outcome after TAVR.

### Outlook

We think that both approaches, MAC and LA, will have a role in the future. In addition to the patient factors, there will be center and operator experience and local logistics which may play in the decision-making. Patient factors need to be defined, those at high risk for GA (for example, severe lung disease) may be better treated with MAC. In particular, also, the patient preference for MAC or GA will have to be taken into consideration. For now, the decision should be made by a “heart team” which also includes a cardiac anesthesiologist together with the patient.

### Limitations

This meta-analysis is based on seven non-randomized studies exclusively. The results are therefore subject to confounding factors, mainly based on a learning curve effect, and the assignment to GA or MAC is often based on patients’ co-morbidities.

Moreover, variations in the training might have had an impact on the choice of anesthesia used, as Medtronic encourages more MAC, compared to Edwards training, which is in favor of GA.

One study retrieved data from the large CoreValve ADVANCE registry [5]. These data were presented as an abstract only but have not been published in the peer reviewed literature so far. Most centers start a TAVR program using GA and switch to MAC after they have become experienced, which additionally contributes to the heterogeneity of the studies. Most of the studies are relatively small and the studies are rather heterogeneous, which may generate false negative results. Interestingly, in the CoreValve ADVANCE registry, which only involves higher volume operators (>40 TAVRs), no mortality benefit was seen for MAC [41].

It is important to recognize that patients who are selected for MAC were maybe chosen because they were expected to be less at risk of technical complications or in need for additional imaging, such as TEE? Although the baseline characteristics of the two groups suggest that they are similar in this aspect, certain indicators for challenging procedures, such as aortic valve and root anatomy, and others on the general condition of patients, such as frailty or immobilization, have not been assessed in the analyzed studies. Therefore, there may be a selection bias for MAC patients, which could not be discriminated in this meta-analysis.

This was a study-level meta-analysis. An individual patient data analysis may provide further insights. Endpoint definitions were not uniform, which contributes to the heterogeneity among the different studies. Also, the published relative risk ratio on 30-day mortality varied widely among the included studies, which makes a uniform interpretation difficult. Indeed, only a large scale randomized trial would be powered to allow for reliable validation of MAC in TAVR.

## Conclusions

The results of this meta-analysis demonstrate that there is no significant difference in outcomes using either MAC or GA for TAVR procedures. While GA can have advantages, including improved peri-procedural imaging, MAC may be associated with reduced procedural time and shorter hospital stay. Randomized or large scale observational studies from national registries are now needed to identify those patients who may truly benefit from this approach and to define the circumstances under which it should be considered.

## Abbreviations

GA: General anesthesia; MAC: Local anesthesia with monitored anesthesia care; PRISMA: Preferred Reporting Items for Systematic Reviews and Meta-Analyses; RR: Risk ratio; TAVR: Transcatheter aortic valve replacement; TEE: Transesophageal echocardiography.

## Competing interest

None of the authors have a conflict of interest to declare.

## Authors’ contributions

GMF and PM had the idea for the study, collected data and drafted the manuscript. AJL, JW, MR, ST and MR have made substantial contributions to conception and design, and interpretation of the data, and revised the manuscript critically for important intellectual content. DW was responsible for statistics. NH, OW and DH-S have made substantial contributions to acquisition of data and analysis and interpretation of data, and they have been involved in drafting the manuscript. All authors read and approved the final manuscript.

## Pre-publication history

The pre-publication history for this paper can be accessed here:

http://www.biomedcentral.com/1741-7015/12/41/prepub

## Supplementary Material

Additional file 1 Checklist of preferred reporting items for systematic reviews and meta-analyses (PRISMA). Click here for file

Additional file 2 Search syntax for Medline. Click here for file

Additional file 3 Endpoint definitions of the included studies. Click here for file

Additional file 4 Valve types used in the individual studies. Click here for file

Additional file 5 Decision-making process in individual studies regarding use of general or local anesthesia. Click here for file

Additional file 6 **Forest plot of risk ratios for 30-day cardiac and procedure-related mortality.** CI: confidence interval. Click here for file

Additional file 7 Reasons for the conversion from local to general anesthesia. Click here for file

Additional file 8 **Forest plot of risk ratios (RR) for vascular complications.** CI: confidence interval. Click here for file

Additional file 9 **Forest plot of risk ratios for procedural success.** CI: confidence interval. Click here for file

Additional file 10 Forest plot of risk ratios for myocardial infarction CI: confidence interval. Click here for file

Additional file 11 Forest plot of risk ratios for acute kidney injury CI: confidence interval. Click here for file

Additional file 12 **Forest plot of risk ratios for sepsis.** CI: confidence interval. Click here for file
