# Supplementary material for: Local versus general anesthesia for transcatheter aortic valve implantation (TAVR) – systematic review and meta-analysis
Source: BMC Med. 2014 Mar 10;12:41. doi: 10.1186/1741-7015-12-41 (PMC4022332; doi:10.1186/1741-7015-12-41)
Supplement: Additional file 2 — Search syntax for Medline. [file 1741-7015-12-41-S2.doc]

| Search | # of abstracts |
| --- | --- |

Search History

Limits: Humans, Randomized Controlled Trial,

#10 #9 AND #4 81

#9 #7 AND #8 64683

#8 "Anesthesia, general"[MeSH Terms] 47530

#7 #5 OR #6 19544

#6 "Anesthesia, Local"[MESH] 13664

## #5 “conscious sedation”[MESH] 2163

#4 #1&#2 590

#3 “TAVI” [All fields] 811

#2 Search "aortic valve"[MESH] 20130

#1 Search "transcatheter "[MESH] 9990

Supplementary File 2: Search strategy for MEDLINE (search date September 10, 2013).
