# Supplementary material for: Local versus general anesthesia for transcatheter aortic valve implantation (TAVR) – systematic review and meta-analysis
Source: BMC Med. 2014 Mar 10;12:41. doi: 10.1186/1741-7015-12-41 (PMC4022332; doi:10.1186/1741-7015-12-41)
Supplement: Additional file 3 — Endpoint definitions of the included studies. [file 1741-7015-12-41-S3.doc]

**Supplementary File 3.** Endpoint definitions of the included trials.

| **Study** |  | |  |  | **Primary endpoint** | | **Other endpoints** |  |  |  |  | |
| --- | --- | --- | --- | --- | --- | --- | --- | --- | --- | --- | --- | --- |
| Yamamoto |  | |  |  | 30-day mortality | | Procedural time, total hospital stay, conversion from MAC to GA, post-operative complications (Valve Academic Research Consortium criteria*) |  |  |  |  | |
| Motloch |  | |  |  | 30-day mortality | | Procedure time (pre-medication with midazolam until transfer to ICU) length of hospital stay,  post procedural complications, calculation of labor costs |  |  |  |  | |
| Dhedin 30-day mortality Procedure duration, hospital stay, post-operative complications | | | | | | | | | | |  | |
| Ben-Dor In-hospital death Procedure duration, in-hospital stay, post-operative complications  30-day mortality | | | | | | | | | | |  |  |
| Behan |  |  | |  | 30-day mortality | Procedure duration, in-hospital stay, post-operative complications | |  |  |  |  |  |
| Linke |  |  | |  | 30-day mortality | In-hospital stay, post-operative complications, MACCE | |  |  |  |  |  |
| Covello |  |  | |  | 30-day mortality | In-hospital stay , conversion from MAC to GA, post-operative complications | |  |  |  |  |  |
|  |  | |  |  |  |  | |  |  |  |  | |

* Leon MB, Piazza N, Nikolsky E, Blackstone EH, Cutlip DE, Kappetein AP, Krucoff MW, Mack M, Mehran R, Miller C, Morel MA, Petersen J, Popma JJ, Takkenberg JJ, Vahanian A, van Es GA, Vranckx P, Webb JG, Windecker S, Serruys PW. Standardized endpoint definitions for transcatheter aortic valve implantation clinical trials: a consensus report from the Valve Academic Research Consortium. Eur Heart J 2011;32:205e217.
