# Supplementary material for: Local versus general anesthesia for transcatheter aortic valve implantation (TAVR) – systematic review and meta-analysis
Source: BMC Med. 2014 Mar 10;12:41. doi: 10.1186/1741-7015-12-41 (PMC4022332; doi:10.1186/1741-7015-12-41)
Supplement: Additional file 4 — Valve types used in the individual studies. [file 1741-7015-12-41-S4.docx]

**Supplementary file 4.** Valve type.

| **Study** | **Edwards**  **Sapien** | **Medtronic**  **CoreValve** |  |
| --- | --- | --- | --- |
| Yamamoto | 14 | 160 |  |
| Motloch | 74 | 0 |  |
| Dhedin | NA | NA |  |
| Ben-Dor | 92 | 0 |  |
| Behan | 0 | 12 |  |
| Linke | 0 | 996 |  |
| Covello | 46 | 23 |  |

NA: not available
