# Supplementary material for: Local versus general anesthesia for transcatheter aortic valve implantation (TAVR) – systematic review and meta-analysis
Source: BMC Med. 2014 Mar 10;12:41. doi: 10.1186/1741-7015-12-41 (PMC4022332; doi:10.1186/1741-7015-12-41)
Supplement: Additional file 5 — Decision-making process in individual studies regarding use of general or local anesthesia. [file 1741-7015-12-41-S5.docx]

**Supplementary file 5: Reasons for use of GA versus LA**

| **Study** | **Reason for GA or LA** |
| --- | --- |
| Yamamoto | During the early phase of the physician learning curve for TAVI from December 2007 to December 2009, GA was mainly used because the operator considered the requirement for transesophageal echocardiography during the procedure important. After this phase, from January 2010 to December 2011, most patients underwent TAVI under LACS. In 2011, 1 patient started TAVI under GA because of peripheral artery disease and the possibility of a switch to a nontransfemoral approach during the procedure. |
| Motloch | In the ﬁrst cohort of 33 consecutive patients, TAVI was performed under GA followed by a second cohort of 41 patients who underwent TAVI under LAPS |
| Dhedin | NA |
| Ben-Dor | During the initial experience, most of the procedures were done under GA but in later stages most of the procedures were done under LA. |
| Behan | The ﬁrst three patients had the procedure done under GA, practice was then switched to LA after one of the first patients had a GA-related  complication. |
| Linke | NA (published as abstract only) |
| Covello | GA was mostly performed at the beginning of the operator`s learning curve and in patients who require surgical vascular access or who were at high risk for major vascular complications, in severely obese patients, in patients with congestive heart failure and in those who were restless. |

TAVI: transcatheter aortic valve implantation. GA: general anesthesia. LA: local anesthesia.NA: information not available.
