# Supplementary figures and images for: Local versus general anesthesia for transcatheter aortic valve implantation (TAVR) – systematic review and meta-analysis
Source: BMC Med. 2014 Mar 10;12:41. doi: 10.1186/1741-7015-12-41 (PMC4022332; doi:10.1186/1741-7015-12-41)

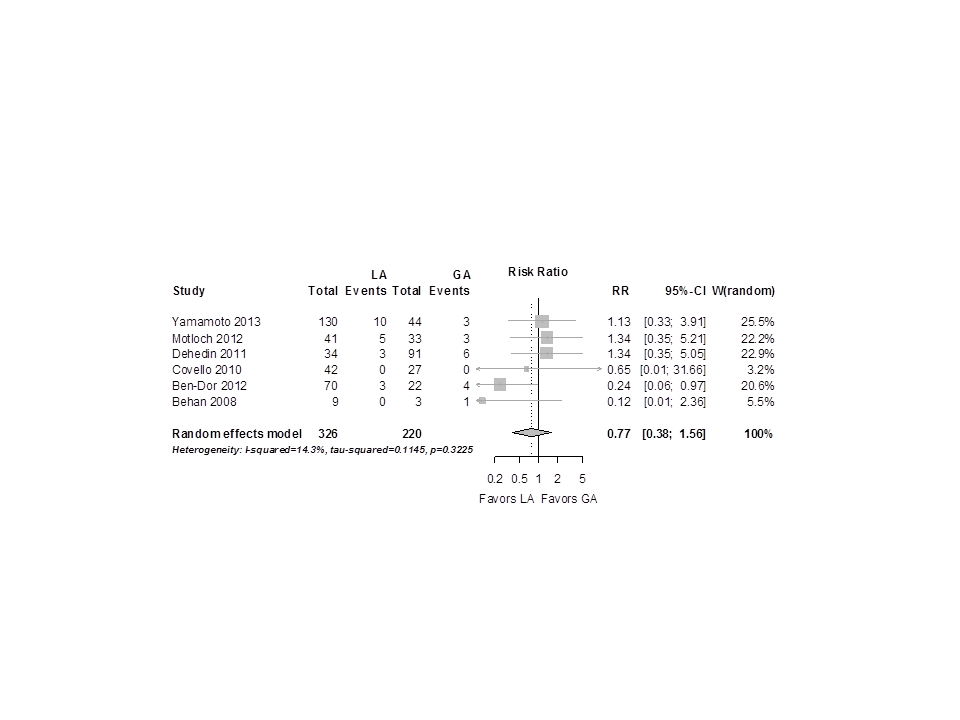

Supplement: Additional file 6 — Forest plot of risk ratios for 30-day cardiac and procedure-related mortality. CI: confidence interval. [file 1741-7015-12-41-S6.tiff]

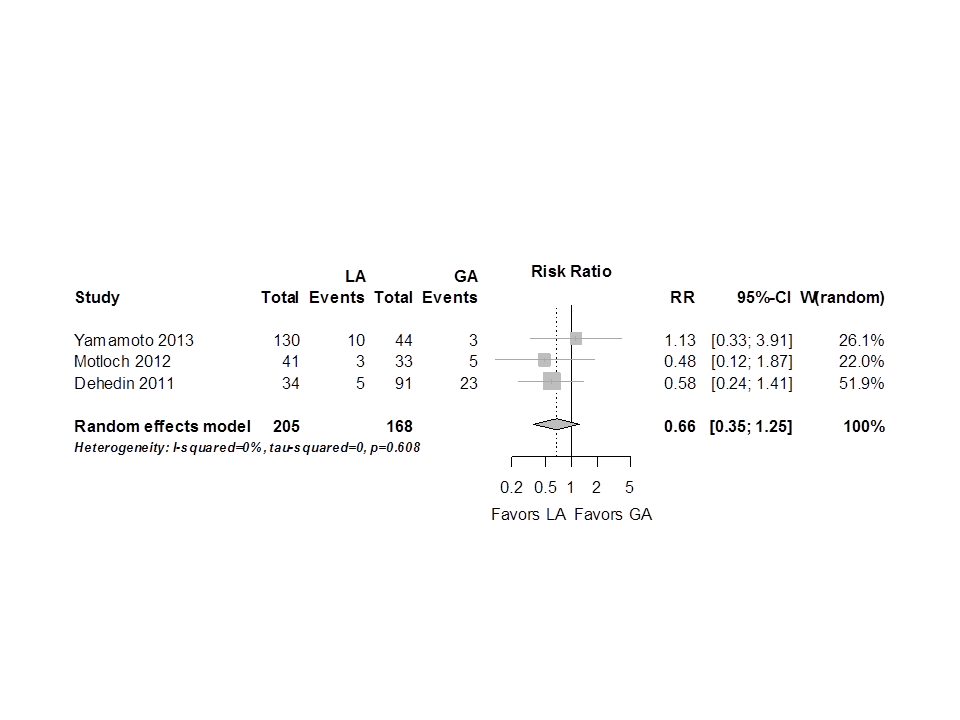

Supplement: Additional file 8 — Forest plot of risk ratios (RR) for vascular complications. CI: confidence interval. [file 1741-7015-12-41-S8.tiff]

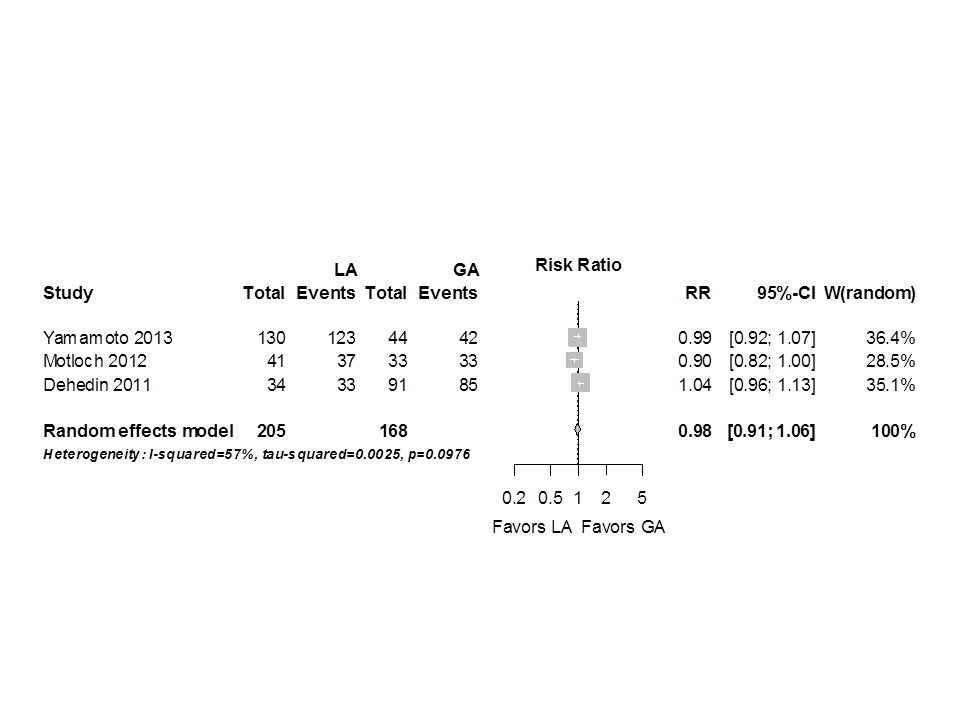

Supplement: Additional file 9 — Forest plot of risk ratios for procedural success. CI: confidence interval. [file 1741-7015-12-41-S9.tiff]

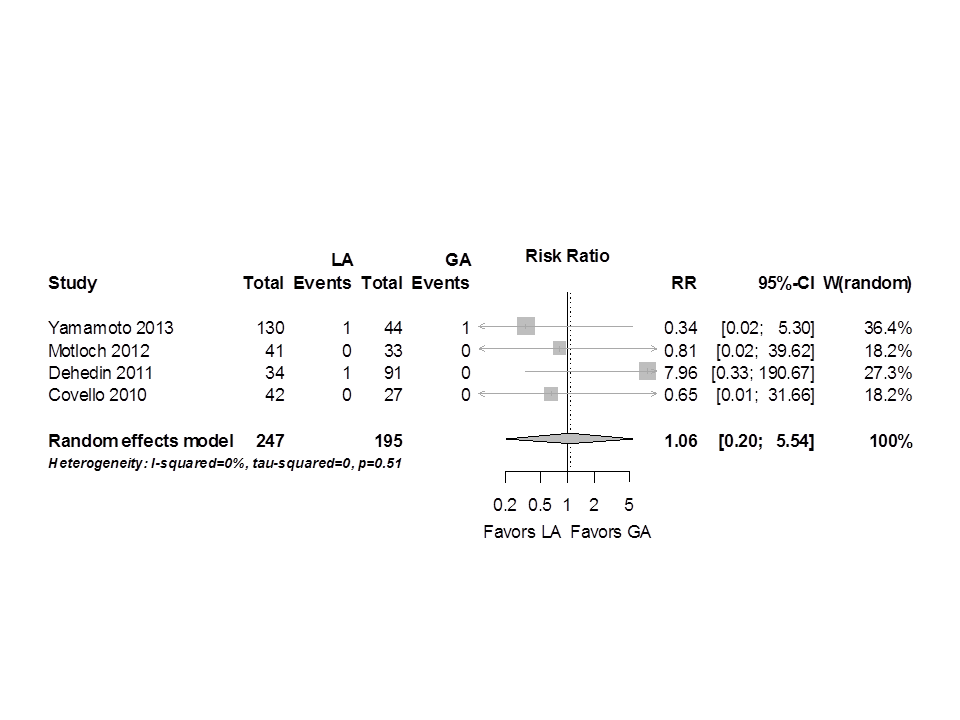

Supplement: Additional file 10 — Forest plot of risk ratios for myocardial infarction CI: confidence interval. [file 1741-7015-12-41-S10.tiff]

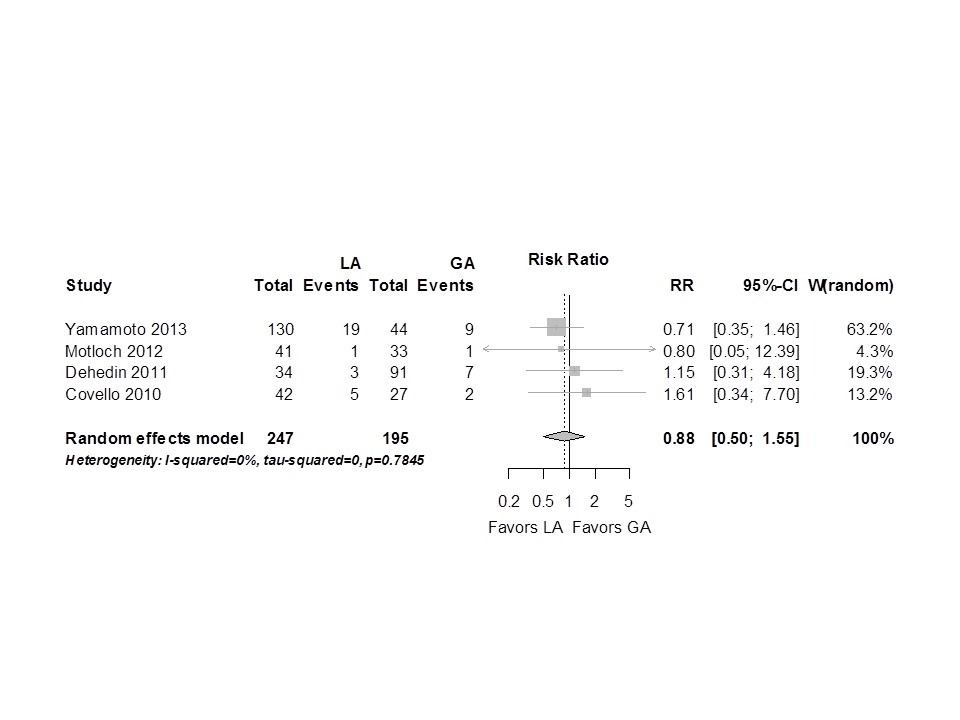

Supplement: Additional file 11 — Forest plot of risk ratios for acute kidney injury CI: confidence interval. [file 1741-7015-12-41-S11.tiff]

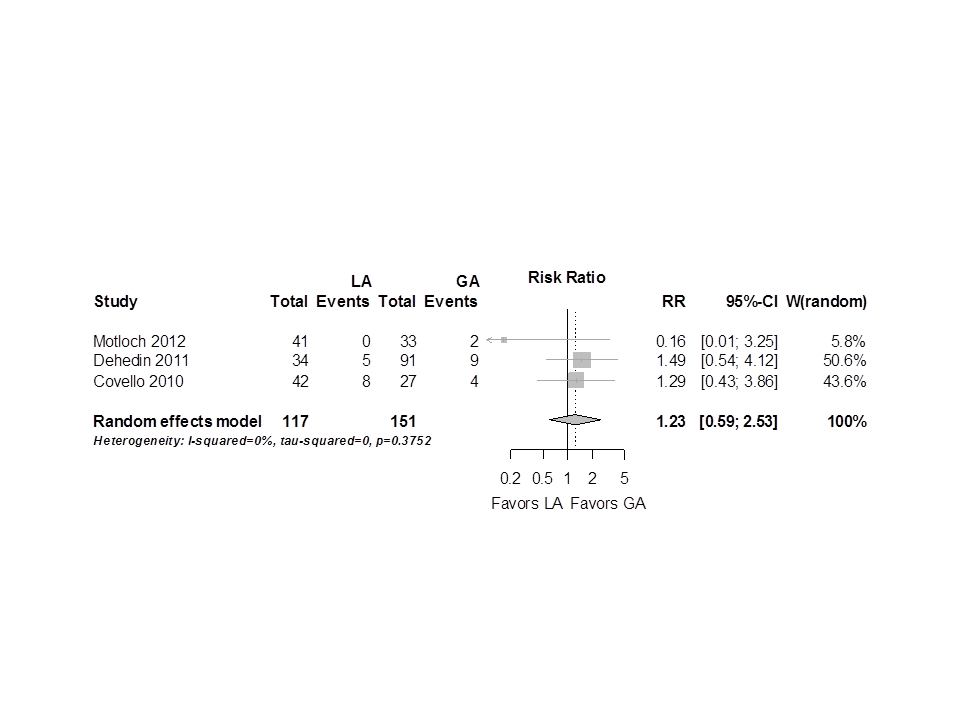

Supplement: Additional file 12 — Forest plot of risk ratios for sepsis. CI: confidence interval. [file 1741-7015-12-41-S12.tiff]
