# Supplementary material for: Local versus general anesthesia for transcatheter aortic valve implantation (TAVR) – systematic review and meta-analysis
Source: BMC Med. 2014 Mar 10;12:41. doi: 10.1186/1741-7015-12-41 (PMC4022332; doi:10.1186/1741-7015-12-41)
Supplement: Additional file 7 — Reasons for the conversion from local to general anesthesia. [file 1741-7015-12-41-S7.docx]

**Supplementary file 10.** Characteristics of patients with conversion from LA to GA.

| **Reason for conversion** | **Number of patients** | **30-day mortality** |
| --- | --- | --- |
| Cardiac tamponade | 2 | 1 |
| Cardiac arrest | 3 | 1 |
| Hemodynamic compromise | 2 | 0 |
| Stroke | 1 | 1 |
| Myocardial infarction | 1 | 1 |
| Non-compliance | 2 | 0 |
| Respiratory failure | 3 | 0 |
| Other | 4 | 0 |

Based on 251 patients (in all investigated studies) undergoing LA
